# Revealing Two Distinct Formation Pathways of 2D Wurtzite‐CdSe Nanocrystals Using In Situ X‐Ray Scattering

**DOI:** 10.1002/advs.202307600

**Published:** 2023-12-10

**Authors:** Hyo Cheol Lee, Megalamane S. Bootharaju, Kyunghoon Lee, Hogeun Chang, Seo Young Kim, Eonhyoung Ahn, Shi Li, Byung Hyo Kim, Hyungju Ahn, Taeghwan Hyeon, Jiwoong Yang

**Affiliations:** ^1^ Department of Energy Science and Engineering Daegu Gyeongbuk Institute of Science and Technology (DGIST) Daegu 42988 Republic of Korea; ^2^ Center for Nanoparticle Research Institute for Basic Science (IBS) Seoul 08826 Republic of Korea; ^3^ School of Chemical and Biological Engineering and Institute of Chemical Processes Seoul National University Seoul 08826 Republic of Korea; ^4^ Samsung Advanced Institute of Technology Samsung Electronics Suwon 16678 Republic of Korea; ^5^ Department of Material Science and Engineering Soongsil University Seoul 06978 Republic of Korea; ^6^ Pohang Accelerator Laboratory Pohang 37673 Republic of Korea; ^7^ Energy Science and Engineering Research Center Daegu Gyeongbuk Institute of Science and Technology (DGIST) Daegu 42988 Republic of Korea

**Keywords:** 2D nanocrystals, formation mechanism, in situ small‐angle X‐ray scattering, nanoribbons, nanosheets, quantum‐sized semiconductor nanocrystals

## Abstract

Understanding the mechanism underlying the formation of quantum‐sized semiconductor nanocrystals is crucial for controlling their synthesis for a wide array of applications. However, most studies of 2D CdSe nanocrystals have relied predominantly on ex situ analyses, obscuring key intermediate stages and raising fundamental questions regarding their lateral shapes. Herein, the formation pathways of two distinct quantum‐sized 2D wurtzite‐CdSe nanocrystals — nanoribbons and nanosheets — by employing a comprehensive approach, combining in situ small‐angle X‐ray scattering techniques with various ex situ characterization methods is studied. Although both nanostructures share the same thickness of ≈1.4 nm, they display contrasting lateral dimensions. The findings reveal the pivotal role of Se precursor reactivity in determining two distinct synthesis pathways. Specifically, highly reactive precursors promote the formation of the nanocluster‐lamellar assemblies, leading to the synthesis of 2D nanoribbons with elongated shapes. In contrast, mild precursors produce nanosheets from a tiny seed of 2D nuclei, and the lateral growth is regulated by chloride ions, rather than relying on nanocluster‐lamellar assemblies or Cd(halide)_2_–alkylamine templates, resulting in 2D nanocrystals with relatively shorter lengths. These findings significantly advance the understanding of the growth mechanism governing quantum‐sized 2D semiconductor nanocrystals and offer valuable guidelines for their rational synthesis.

## Graphical abstract

Two critical pathways of two distinct quantum‐sized 2D wurtzite‐CdSe nanocrystals are revealed by the combined approach using various characterization techniques. This study provides rational explanations for intermediate stages, the difference in the laterals dimensions, and the origin of different synthesis pathways of quantum‐sized 2D semiconductor nanocrystals.

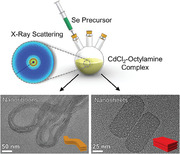

## 1 Introduction

Over the last several decades, there have been tremendous efforts toward the size‐ and shape‐controlled synthesis of colloidal nanocrystals,^[^ ^1^, ^2^, ^3^, ^4^, ^5^, ^6^, ^7^, ^8^, ^9^ ^]^ especially quantum‐sized semiconductor nanocrystals with 0D,^[^ ^10^, ^11^, ^12^, ^13^, ^14^ ^]^ 1D,^[^ ^14^, ^15^, ^16^, ^17^, ^18^, ^19^, ^20^ ^]^ and 2D^[^ ^21^, ^22^, ^23^, ^24^, ^25^, ^26^, ^27^, ^28^ ^]^ shapes. Among these, quantum‐sized 2D semiconductor nanocrystals are of particular interest owing to their unique optical properties, derived from atomically uniform thickness, such as an extremely narrow emission linewidth of ≈10 nm and high photoluminescence (PL) quantum yield,^[^ ^29^ ^]^ which are difficult to attain for conventional 0D quantum dots. Consequently, 2D nanocrystals have been utilized in light‐emitting diodes,^[^ ^30^, ^31^, ^32^ ^]^ lasers,^[^ ^33^, ^34^, ^35^ ^]^ photocatalyst,^[^ ^36^ ^]^ and sensors.^[^ ^37^, ^38^ ^]^ Since the first report on quantum‐sized 2D CdSe nanoribbons,^[^ ^23^ ^]^ synthesis protocols have expanded to encompass other 2D semiconductors, such as CdS,^[^ ^24^ ^]^ CdTe,^[^ ^25^, ^39^, ^40^ ^]^ ZnSe,^[^ ^41^ ^]^ ZnS,^[^ ^22^ ^]^ CuS,^[^ ^22^ ^]^ PbS,^[^ ^26^ ^]^ perovskite,^[^ ^42^, ^43^ ^]^ and their heterostructures.^[^ ^31^, ^32^, ^33^, ^34^, ^35^, ^44^ ^]^ They are designated according to their lateral shapes, such as nanoribbons,^[^ ^23^ ^]^ nanosheets,^[^ ^25^ ^]^ nanoplatelets,^[^ ^45^ ^]^ nanohelices,^[^ ^46^ ^]^ quantum disks,^[^ ^47^ ^]^ or quantum nets.^[^ ^48^ ^]^ Considering the crystal structures of II–VI semiconductor materials, i.e., cubic zincblende and hexagonal wurtzite, the formation of 2D nanocrystals are inherently challenging,^[^ ^49^, ^50^, ^51^ ^]^ suggesting that comprehensive understanding of their formation mechanism is required.

The formation mechanism of 2D zincblende‐CdSe nanoplates has been studied extensively, and several hypotheses have been made, including self‐assembly,^[^ ^52^ ^]^ soft templating,^[^ ^47^ ^]^ continuous lateral growth,^[^ ^40^, ^45^, ^53^, ^54^, ^55^, ^56^ ^]^ and phase‐transition.^[^ ^57^ ^]^ Despite the early synthesis of 2D wurtzite‐CdSe nanocrystals, research on their formation mechanism significantly lags behind that of their cubic counterparts. It has been suggested that the formation of 2D wurtzite‐CdSe nanocrystals is induced by the soft templates formed by the reaction intermediates. For instance, magic‐sized nanoclusters^[^ ^58^, ^59^, ^60^, ^61^, ^62^, ^63^, ^64^, ^65^, ^66^, ^67^, ^68^ ^]^ (e.g., (CdSe)_13_ and (CdSe)_34_)^[^ ^58^, ^59^, ^60^, ^61^, ^62^, ^63^, ^64^, ^65^, ^66^ ^]^ have been suggested to form 2D lamellar assemblies, which crystallize to form 2D nanocrystals.^[^ ^24^, ^58^ ^]^ In another example, it has been proposed that the formation of 2D lamellar soft templates by metal halide–alkylamine precursor complexes induces 2D growth of wurtzite nanosheets.^[^ ^25^, ^59^ ^]^ However, they cannot well elucidate the formation mechanism while considering various synthesis protocols and different lateral sizes and shapes of the final nanocrystals. Furthermore, most of the previous works relied on ex situ measurements, which could not reveal the intermediate stages of the growth process clearly (e.g_._, how and which lamellar structures govern 2D crystallization and influence the lateral size of final products). Hence, real‐time observation is necessary to comprehend the complete growth mechanism.

Recently, in situ small‐angle X‐ray scattering (SAXS) has emerged as one of the most effective techniques for studying nanocrystal formation mechanism, because this nondestructive method can trace subnanometer spatial changes with a high temporal resolution of a few microseconds.^[^ ^69^ ^]^ The formation mechanism and shape evolution of metal nanocrystals,^[^ ^70^ ^]^ semiconductor nanocrystals,^[^ ^55^, ^56^, ^71^, ^72^, ^73^ ^]^ and nanoclusters^[^ ^60^, ^74^, ^75^ ^]^ have been extensively studied using SAXS. For 2D nanocrystals, in situ SAXS has been used to explore the lamellar templated growth of Cu_2‐_ *_x_* S,^[^ ^72^ ^]^ self‐assembly of CdSe–460‐nm clusters into cubic 2D nanoplatelets,^[^ ^73^ ^]^ and lateral growth of cubic‐CdSe nanoplatelets.^[^ ^55^, ^56^ ^]^ However, to our knowledge, real‐time observations on the growth of 2D wurtzite‐CdSe nanocrystals have been limited to the generation of nanocluster assemblies in the initial stages of synthesis.^[^ ^61^ ^]^

Herein, we investigated the nucleation and growth processes of two representative quantum‐sized 2D wurtzite‐CdSe nanocrystals using in situ SAXS, transmission electron microscopy (TEM), and absorption spectroscopy. The results show that these two different kinds of 2D CdSe nanocrystals exhibit two distinct synthesis pathways. Nanoribbons form after magic‐sized (CdSe)_13_ clusters assembled into large‐sized 2D lamellar structures, disclosing the dynamics of the intermediate states. Whereas, the synthesis of nanosheets can bypass the formation of nanocluster‐lamellar assemblies, and the Cd(halide)_2_–alkylamine templates dissolve prior to 2D growth, which contradicts the previous reports based on ex situ studies. Cl^−^ ions facilitate the 2D growth of nanosheets along the lateral direction—which is typically difficult to control—may be readily tuned by adjusting the number of Cl^−^ anions. The Se precursor reactivity has a critical role in determining synthesis pathways, by dictating the generation of nanoclusters during the initial growth stage of 2D nanocrystals. This study enhances fundamental understanding of the formation mechanism of 2D nanocrystals and provides a simple and generic approach to controlling the lateral dimension of 2D nanocrystals.

## 2 Results and Discussion

**Figure 1:**
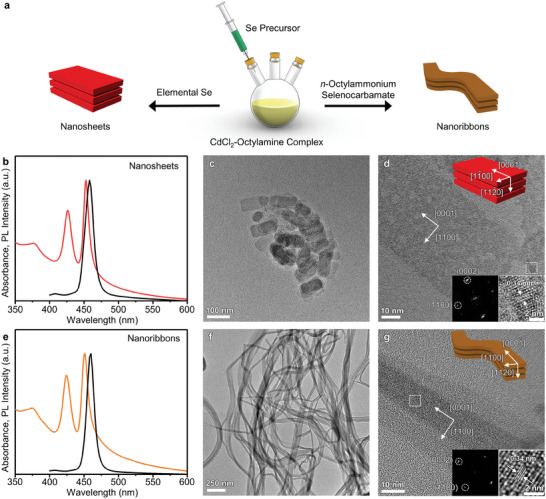
Synthesis and characterization of 2D CdSe nanosheets and nanoribbons. a) Schematic illustration showing synthesis of 2D CdSe nanosheets (left) and 2D CdSe nanoribbons (right). b) Absorption (red) and PL (black) spectra and c,d) TEM images of 2D CdSe nanosheets. e) Absorption (orange) and PL (black) spectra and f,g) TEM images of 2D CdSe nanoribbons. The upper‐right insets in panels (d) and (g) show the nanocrystal structures and crystal orientation. The lower‐right insets in panels (d) and (g) show magnified TEM images of the white‐dotted rectangular areas, showing the lattice spacings and the corresponding FFT patterns.

Two types of representative quantum‐sized 2D wurtzite‐CdSe nanocrystals,^[^ ^23^, ^25^ ^]^ known as nanosheets and nanoribbons, were selected as model systems (**Figure** **1**). Depending on the Se precursor, either nanosheets or nanoribbons were produced (see Experimental Section). Specifically, nanosheets were formed using elemental Se in *n*‐octylamine (OcAm), whereas the use of *n*‐octylammonium selenocarbamate produced nanoribbons (Figure 1a). Both nanocrystals exhibit heavy and light hole‐excitonic transitions and extremely sharp emission spectra (Figure 1b,e, full‐width at half‐maximum: ≈12 nm), which are characteristics of 2D CdSe nanocrystals. Although nanosheets and nanoribbons possess the same thickness (≈1.4 nm, Figure S1, Supporting Information) and wurtzite crystal structure,^[^ ^23^, ^25^ ^]^ their lateral shapes are vastly different (Figure 1c,f; Figure S2, Supporting Information), raising fundamental questions about their formation pathways. Nanosheets are rectangular with a moderate aspect ratio (width: ≈50 nm, length: ≈100 nm), whereas nanoribbons are wider (≈80 nm) and considerably longer (a few micrometers). They share the same planar lattice direction, [$1\bar{1}00$] along the width and [$000\bar{1}$] along the length,^[^ ^23^, ^25^ ^]^ as evidenced by TEM images and Fast Fourier Transform (FFT) analysis (Figure 1d,g). Both form lamellar structures by repeatedly stacking 2D nanocrystals (Figure S3, Supporting Information).

**Figure 2:**
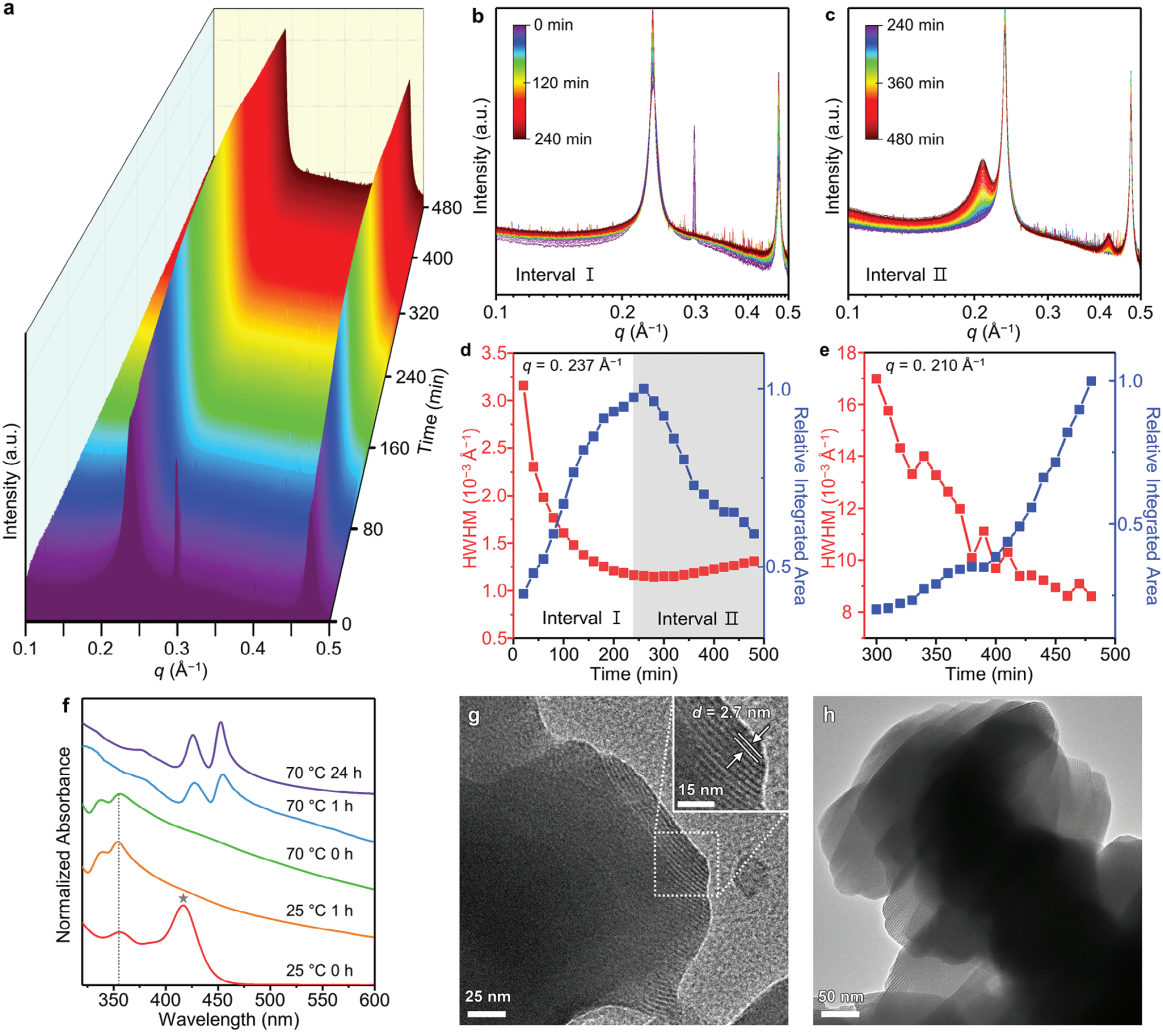
Formation of CdSe nanoribbons. a) In situ SAXS patterns depicting the evolution of CdSe nanoribbons, b) the first half of the reaction (0–4 h), and c) the latter half of the reaction (4–8 h). Relative integrated area and linewidth (half‐width at half‐maximum, HWHM) for the first‐order peaks of d) the cluster assemblies (*q* = 0.237 Å^−1^) and e) the nanoribbon assemblies (*q* = 0.210 Å^−1^) as a function of reaction time. A maximum area value during the observation is set as 1. The scattering intensity of all panels is plotted using a logarithmic scale except for panel (d) and (e) (linear scale). f) Temporal evolution of absorption spectra of a series of aliquots during nanoribbon synthesis. After 1 h of reaction at room temperature (25 °C), the reaction temperature was increased to 70 °C. The position of band‐edge transitions of magic‐sized (CdSe)_34_ and (CdSe)_13_ clusters are depicted by a gray star and gray dotted line, respectively. g,h) TEM images of (CdSe)_13_ cluster assemblies. The ordered region with a striped pattern represents the lamellar structure. Inset in panel (g) shows the magnified image.

In situ synchrotron SAXS was conducted to understand the 2D growth mechanism (for experimental details, see Figure S4, Supporting Information and Experimental Section). The temporal evolution of scattering patterns of reaction mixtures was monitored in real‐time. **Figure** **2**a and Figures S5 and S6 (Supporting Information) show the in situ SAXS profiles during CdSe nanoribbon formation. The profiles of the initial mixture exhibit a series of first‐ and higher‐order reflections originating from lamellar assemblies of (CdSe)_13_ clusters, indicating that magic‐sized (CdSe)_13_ clusters form and assemble into 2D soft templates in the initial reaction mixture (Figure 2b). The position (*q*) of the first‐order peak (≈0.237 Å^−1^) corresponds to interlayer spacing (*d*) of ≈2.65 nm, which is consistent with ex situ SAXS measurements of (CdSe)_13_ clusters (Figure S7, Supporting Information).^[^ ^58^ ^]^ The sharp reflection peak at ≈0.30 Å^−1^ is related to the unreacted Se precursor, which soon disappeared upon heating (Figure S8, Supporting Information). As the reaction proceeds, an additional series of reflections emerged (Figure 2c). The position of the first‐order peak is ≈0.210 Å^−1^ (*d*: ≈2.99 nm), which is consistent with ex situ SAXS measurements of lamellar‐assembled CdSe nanoribbons (Figure S9, Supporting Information). Thus, these distinct signals at ≈0.237 and ≈0.210 Å^−1^ correspond to nanocluster and nanoribbon assemblies, respectively, suggesting that nanocluster‐lamellar assemblies convert into assembled nanoribbons.

During the initial reaction stage (0–4 h), the integrated area of the first‐order reflection peak originating from nanocluster‐lamellar assemblies increased, while the linewidth decreased (Figure 2d; Figures S10a and S11a, Supporting Information). This suggests the formation of increasingly ordered‐lamellar assemblies as more clusters assembled. After 4 h, the integrated area of the nanocluster assembly peak decreased, whereas that for the nanoribbon peak increased with decreasing linewidth (Figure 2d,e; Figures S10b, and S11b, Supporting Information). The broadening of the linewidth of the cluster peak became apparent with the formation of nanoribbons, implying that the initially assembled clusters transformed into less ordered‐layered assemblies of nanoribbons. In addition, considering the SAXS data showing that more clusters assembled over time, it is plausible to speculate that more clusters could be incorporated into these lamellar assemblies prior to (and during) the transformation. This process likely contributes to the larger lateral size of the resulting nanoribbons. The formation of the lamellar assemblies stabilizes the nanoclusters,^[^ ^75^ ^]^ resulting in a relatively long incubation time of the nanoribbons.

Figure 2f illustrates the temporal evolution of the absorption spectra during nanoribbon synthesis. Ex situ data were recorded due to the strong light scatterings of the nanoribbons (Figure S12, Supporting Information). After injecting Se precursor, a prominent absorption peak appeared at 410 nm with a small transition edge at 353 nm, which are assigned to magic‐sized (CdSe)_34_ and (CdSe)_13_ clusters (Figure S13, Supporting Information), respectively.^[^ ^58^, ^59^, ^60^, ^61^, ^62^, ^63^, ^64^, ^65^, ^66^ ^]^ After 1 h of vigorous stirring at 25 °C, the peak corresponding to (CdSe)_34_ clusters vanished, and only the peak originating from (CdSe)_13_ clusters remained, suggesting that (CdSe)_34_ clusters transformed into (CdSe)_13_ clusters. An ordered lamellar assembly with a striped pattern was observed in TEM images of (CdSe)_13_ clusters (Figure 2g,h), which is similar to that of nanoribbons in terms of shape. The *d*‐spacing measured by TEM (≈2.7 nm, Figure 2g; Figure S14, Supporting Information) is similar to that obtained from SAXS. In addition, the difference in interlayer distances of nanoribbon (2.99 nm) and cluster assemblies (2.65 nm) is similar to the thickness difference of nanoribbons (1.4 nm) and assembled clusters (1.1 nm). This indicates that both assemblies share a similar structure for organic layers, supporting our proposed mechanism wherein assembled nanoclusters are transformed into nanoribbons. The assemblies already have a lateral length of hundreds of nanometers; thus, this explains the large lateral size of the nanoribbons. As the reaction proceeded, the (CdSe)_13_ absorption peaks disappeared, while sharp and intense peaks corresponding to the nanoribbons emerged (Figure 2f). Meanwhile, free (CdSe)_13_ clusters neither converted into nanoribbons nor dissolved at 70 °C (Figure S15, Supporting Information), demonstrating that maintaining lamellar assemblies of (CdSe)_13_ clusters is essential for the synthesis of nanoribbons. While these results strongly imply that the dissolution of (CdSe)_13_ clusters is not significant by the mild heating, the possibility of their partial dissolution—which can lead the crystals to grow larger and preferentially along the specific facet that offers the most energetically favored growth—cannot be completely excluded.

**Figure 3:**
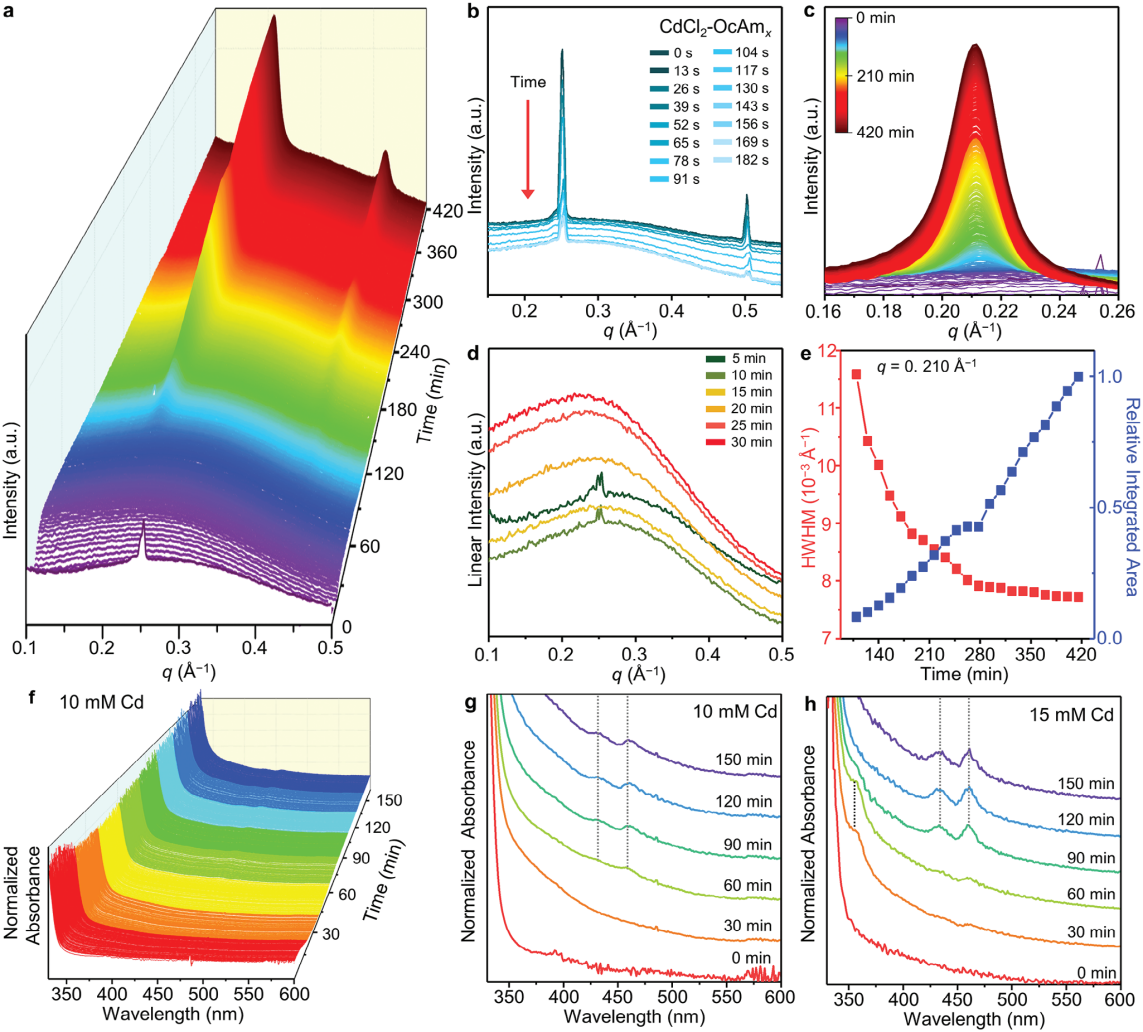
Formation of CdSe nanosheets. a) In‐situ SAXS patterns depicting the evolution of CdSe nanosheets. b) Initial 15 frames (corresponding to 0–3 min) of the SAXS patterns magnified near the regime corresponding to the first and second‐order reflections of the lamellar assemblies of CdCl_2_–OcAm*_x_* complexes. c) Magnified SAXS patterns on the regime corresponding to the first‐order reflections of the lamellar assemblies of nanosheets. d) Representative SAXS curves for the initial 30 min. e) Relative integrated area and linewidth for the first‐order peak of the nanosheet assemblies (*q* = 0.210 Å^−1^) as a function of reaction time. A maximum area value during the observation is set as 1. The scattering intensity of all panels is plotted using a logarithmic scale except for panel (d) and (e) (linear scale). f) In situ absorption spectra depicting the evolution of CdSe nanosheets at the Cd concentration of 10 mm. g) Selected absorption spectra acquired from panel (f). The gray dashed lines indicate the heavy hole‐ and light hole‐excitonic transitions of nanosheets. h) In situ absorption analysis at the higher Cd concentration (15 mm).

Remarkably, the formation pathway of 2D nanosheets differs significantly both from that of the nanoribbons and previously proposed pathways.^[^ ^25^, ^58^ ^]^ **Figure** **3**a and Figure S16 (Supporting Information) depict the time evolution of the in situ SAXS profiles during nanosheet synthesis. Unlike for the nanoribbon synthesis, elemental Se in OcAm was utilized as the Se precursor for nanosheets. A mixture containing CdCl_2_–OcAm*_x_* (0 ≤ *x* ≤ 2) complexes and elemental Se in OcAm was loaded for in situ SAXS (see Experimental Section). Initially, a series of peaks were observed, implying the formation of a lamellar structure of CdCl_2_–OcAm*_x_* complexes. The position (*q*) of the first reflection peak (≈0.250 Å^−1^, *d*: 2.51 nm) corresponds to that of ex situ measurements on purified CdCl_2_–OcAm*_x_* complexes in OcAm (Figure S17, Supporting Information). Previously, soft lamellar templates comprising Cd(halide)_2_–alkylamine complexes have been suggested to induce the 2D growth of CdSe nanocrystals.^[^ ^25^, ^58^ ^]^ However, our in situ SAXS measurements reveal that the lamellar templates of CdCl_2_–OcAm*_x_* complexes dissolve upon heating (Figure 3b). The peak intensity decreased sharply within 3 min, implying that the initial lamellar templates generated by CdCl_2_–OcAm*_x_* were degraded by heating. Thus, an alternative explanation is necessary to explain the 2D growth of CdSe nanosheets.

As the reaction proceeded, a series of peaks originating from the lamellar assemblies of nanosheets emerged. The first‐order reflection peak position was ≈0.210 Å^−1^ (*d*: ≈2.99 nm, Figure 3c), which is almost identical to that of the lamellar assemblies of the nanoribbons owing to their identical thickness (−1.4 nm) and surface ligands (OcAm). This was further confirmed by ex situ SAXS analysis of nanosheets (Figure S18, Supporting Information). However, no peak corresponding to nanocluster‐lamellar assemblies was observed during the nanosheet formation (Figure S19, Supporting Information). The scattering patterns from the initial reaction stage displayed a broad peak at the *q*‐range of ≈0.2–0.3 Å^−1^. An increase in its intensity indicates that polydisperse intermediate species were generated and interacted strongly (Figure 3d). The interactions between those intermediate species gave rise to the strong structure factor. Heterogeneity in particle sizes and shapes was also supported by TEM analysis (Figure S20, Supporting Information). Furthermore, once the lamellar assemblies appear (both for the nanoribbons and the nanosheets), the strong contribution of the structure factor from these assemblies arose. These obstacles made it challenging to extract meaningful form factors such as size and polydispersity under our experimental conditions. Hence, we interpreted the SAXS peaks phenomenologically, based on their *q*‐positions. A series of peaks corresponding to the lamellar assemblies of nanosheets began to emerge over time, without peaks for nanocluster‐lamellar assemblies. This suggests that CdSe nanosheet synthesis bypasses the formation of nanocluster assemblies. The integrated area of the first‐order peak corresponding to the nanosheets continuously increased while its linewidth decreased until the end of the measurement (Figure 3e). These observations imply that the nanosheets form highly ordered lamellar assemblies as they grow. Growth in the lateral direction would increase the van der Waals interactions between nanosheets, resulting in well‐assembled nanosheets. The generation of nanosheets without nanocluster‐lamellar assemblies was also corroborated by atomic force microscopy (AFM) analysis (Figure S21, Supporting Information). Initially, no specific structures with large domain sizes were detected, indicating bypassing the formation of nanocluster‐lamellar assemblies. As the reaction proceeded, the structures with the large domain emerged.

To supplement SAXS analysis, in situ optical spectroscopy was conducted (Figure 3f–h, Experimental Section). Selected absorption spectra for various reaction times are displayed for two different precursor concentrations (Figure 3g,h). During the initial reaction stage (≈30 min), no specific transition was detected and the absorption profiles remained almost constant. Heavy and light hole‐excitonic transitions^[^ ^23^, ^24^, ^25^, ^58^, ^64^ ^]^ of CdSe nanosheets appeared from 60 min onwards. The signals of these transitions intensified over time, implying the formation of more nanosheets. At the lower precursor concentration, intermediate species such as nanoclusters were not clearly detectable (Figure 3g), whereas their presence became apparent as the precursor concentration increased (Figure 3h; Figure S22, Supporting Information). However, it is unlikely that these intermediates would be directly attached to nanosheets for the growth, considering that the optical signals associated with the intermediates disappeared after appearance of the excitonic transitions of nanosheets. It is more plausible that these intermediates dissolved, subsequently releasing ionic or molecular complexes that can contribute to the growth of the nanosheets. The effect of the precursor concentration on the synthesis pathway is further discussed in the later part.

The SAXS results reveal that the synthesis of wurtzite‐CdSe nanosheets occurs by a different pathway from that of nanoribbons, which involves lamellar assemblies of monodispersed nanoclusters. The formation of nanocluster‐lamellar assemblies is not essential for the nanosheet synthesis and Cd(halide)_2_–alkylamine templates dissolve prior to the synthesis. Recently, anion species have been suggested to play a key role in the 2D growth of CdSe nanocrystals. The addition of small anions, such as acetate, is reported to induce the rapid anisotropic growth of 2D nanocrystals.^[^ ^55^ ^]^ Similarly, Buhro and colleagues demonstrated that anion species induce the coalescence of (CdSe)_13_ clusters to form 2D nanocrystals.^[^ ^39^ ^]^ We postulated that Cl^−^ ions originating from CdCl_2_–OcAm*_x_* precursors can promote 2D growth.

**Figure 4:**
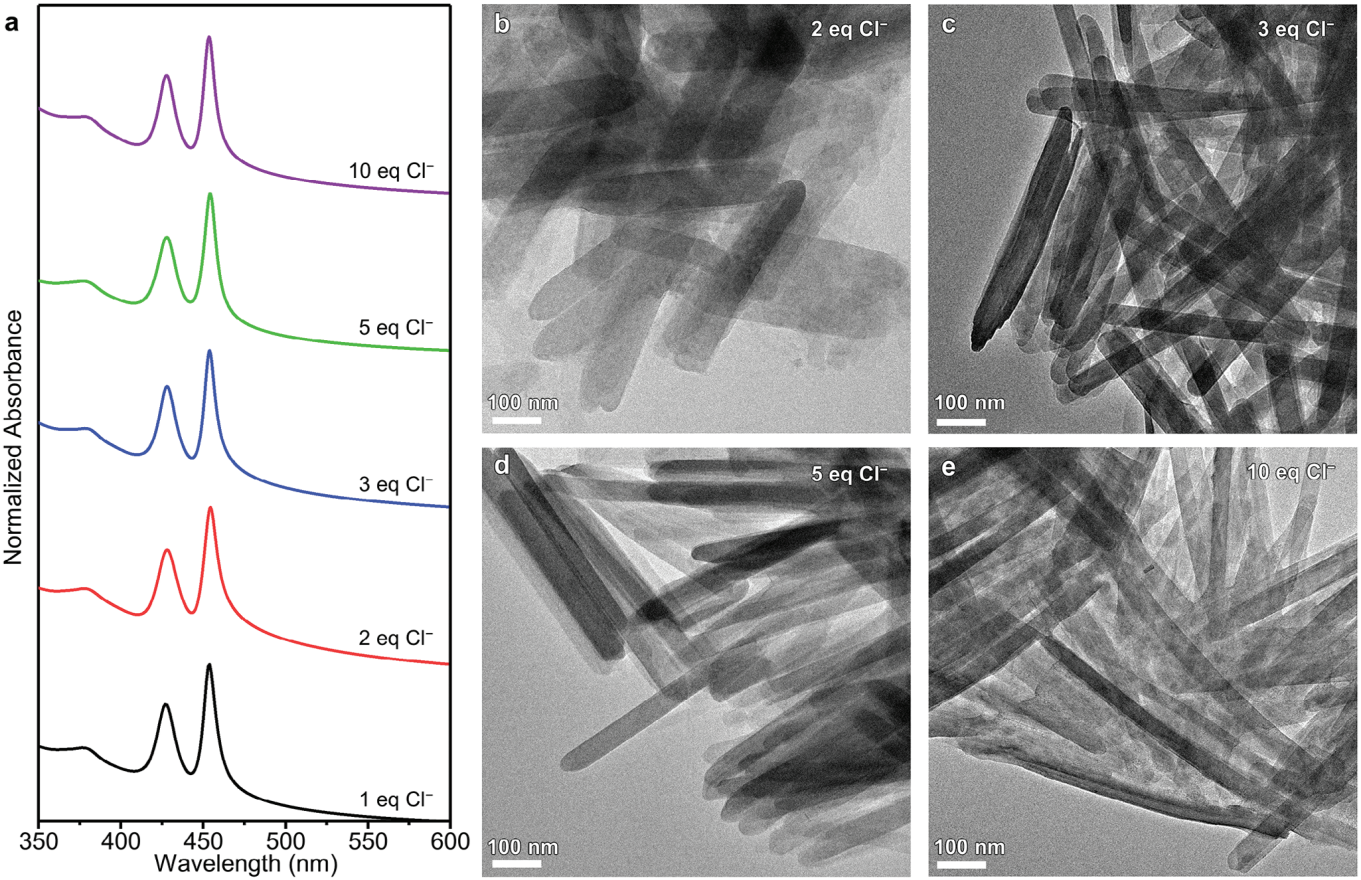
Effect of Cl^−^ ions on nanosheet synthesis. a) Absorption spectra and TEM images of CdSe nanosheets synthesized with b) 2.0, c) 3.0, d) 5.0, and e) 10.0 equivalent Cl^−^ ions. The absorption spectrum of the controlled sample (1.0 equivalent Cl^−^ ions) is presented in panel a for comparison.

To demonstrate the effect of anions on 2D growth, the concentration of Cl^−^ ions was controlled during synthesis by adding *n*‐octylammonium hydrochloride (see Experimental Section and **Figure** **4**). This enables the independent regulation of the number of Cl^−^ ions without interference from the ligand in *n*‐octylammonium hydrochloride because the dissociated ligand is OcAm,^[^ ^76^ ^]^ which is the same as the original ligand and solvent in our experimental conditions. Regardless of the concentration of Cl^−^ ions, the position of the band‐edge transitions in absorption spectra remained identical to that of the original nanosheets (Figure 4a), suggesting that all nanosheets have the same quantum‐confined thickness. Instead, the lateral size of nanosheets became bigger by simply increasing the concentration of Cl^−^ ions and the effect is dominant along polar [$000\bar{1}$] direction (Figure 4b–e). When Cl^−^ ions are introduced, they preferentially bind to the side facets while the basal planes remain passivated by OcAm. This preference is due to the lower average displacement energy for binding sites at the edges compared to those away from the edges.^[^ ^29^ ^]^ Consequently, Cl^−^ ions selectively passivate the small seeds of 2D nanosheets along the side facets, facilitating monomer transport to these facets by reducing the steric barrier. Therefore, the introduction of Cl^−^ ions promotes growth in the lateral rather than the thickness direction. Furthermore, the influence of Cl^−^ ions is more pronounced in the length direction because it aligns with the polar direction [$000\bar{1}$].^[^ ^51^ ^]^ The similar effect was observed for the growth of zincblende 2D nanocrystals in the polar [001] direction.^[^ ^77^ ^]^ Once 2D crystals are formed, they can be stabilized by forming 2D lamellar assemblies with dense ligand passivation on their basal planes.^[^ ^72^ ^]^ This effect was corroborated as 2D crystals cannot maintain their structure at higher temperature (> 160 °C), because the ligand layers lose their close packing (see Experimental Section and Figure S23, Supporting Information).

The fundamental distinction between the two synthesis processes is attributed to the difference in the precursor reactivity. The high reactivity of *n*‐octylammonium selenocarbamate ensures the rapid formation of a large number of magic‐sized (CdSe)_13_ clusters.^[^ ^64^ ^––^ ^66^ ^]^ This induces the formation of nanocluster‐lamellar assemblies, leading to the formation of 2D nanoribbons with large lateral size. In contrast, during the synthesis of nanosheets, the low reactivity of elemental Se does not produce a sufficient amount of (CdSe)_13_ clusters for the formation of nanocluster‐lamellar assemblies, resulting in the synthesis of short nanosheets. It is also consistent with the previous work showing that precursors with high reactivity can stabilize less stable species.^[^ ^78^ ^]^

This hypothesis is further supported by the nanosheet synthesis with various precursor concentrations (Figure 3f–h; Figure S22, Supporting Information). Increasing the precursor concentration promotes the formation of intermediate species owing to the large reactant concentration. The position of the band‐edges of the nanosheets are constant, regardless of the Cd precursor concentrations, indicating that the thickness of the final products is not determined by the reactant concentration under our experimental conditions. This finding is consistent with the fact that the nanosheets and nanoribbons have the same thickness despite the large difference in the precursor reactivity. Furthermore, nanoribbons and nanosheets can be synthesized at the same temperature of 80 °C (Figure S24, Supporting Information). This further supports that the selection of precursors plays a pivotal role in determining the growth pathways and the small temperature difference in their synthesis has relatively minor effects on the overall process.

## 3 Conclusion

**Figure 5:**
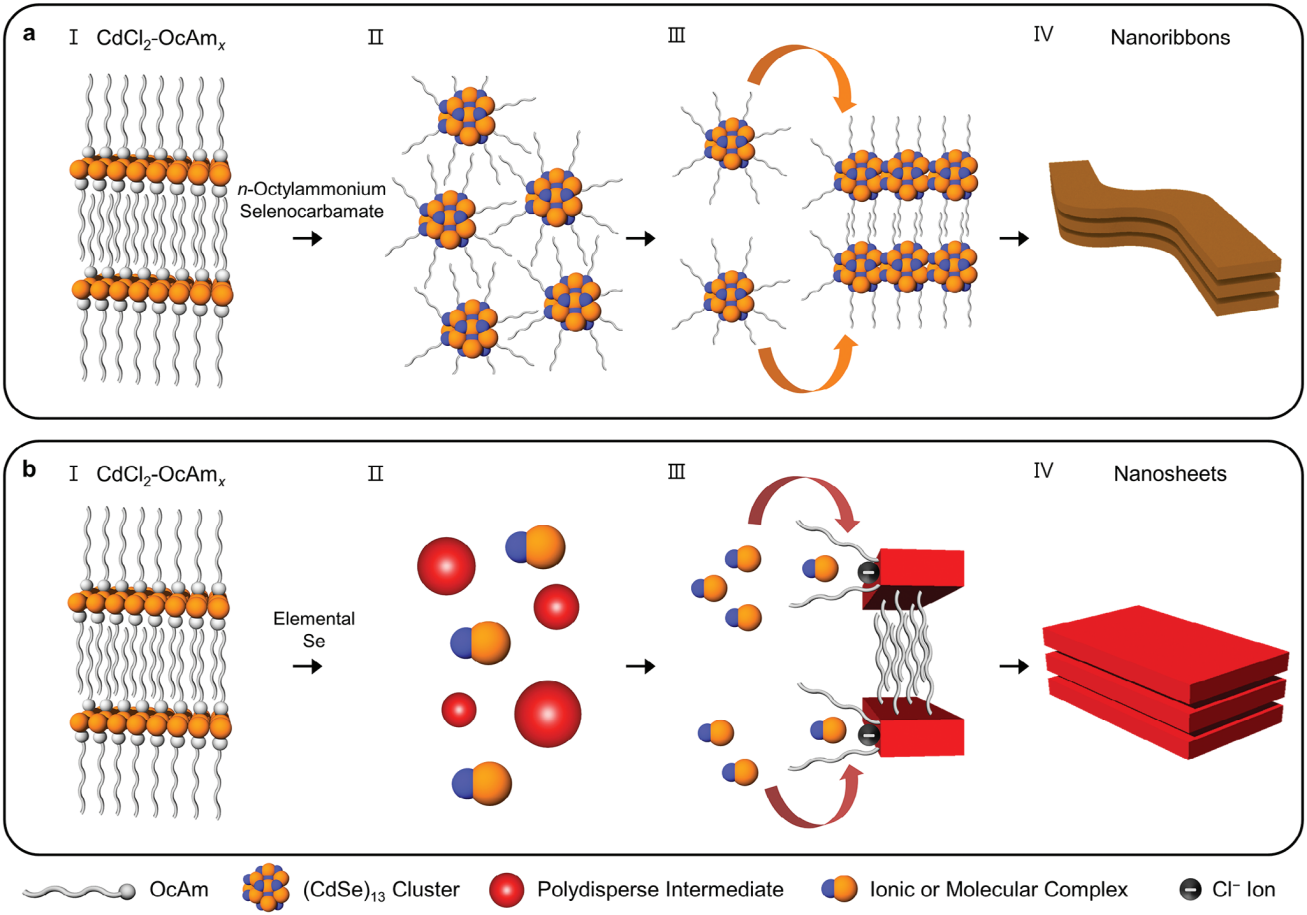
Schematic of the two proposed critical pathways for the synthesis of quantum‐sized 2D CdSe nanocrystals: a) nanoribbons and b) nanosheets. The atoms with orange and blue colors represent Cd and Se atoms, respectively. Please note that the illustrations should be interpreted as conceptual representations of growth pathways for 2D nanocrystals, rather than precise depictions of specific structural details (e.g., the structure of nanoclusters and interdigitation between particles).

In summary, we explore the growth mechanism of two representative quantum‐sized 2D wurtzite‐CdSe nanocrystals. The formation pathway of CdSe nanoribbons is as follows (**Figure** **5**a). 1) CdCl_2_–OcAm*_x_* complexes form lamellar‐assembled structures. 2) Upon introducing *n*‐octylammonium selenocarbamate, magic‐sized (CdSe)_34_ and (CdSe)_13_ clusters are sequentially produced, which then assemble into the 2D lamellar structure. 3) As the reaction progresses, more nanoclusters continue to assemble. 4) The nanoclusters are completely transformed to nanoribbons while preserving their lamellar assemblies. On the other hand, the formation pathway of CdSe nanosheets is different (Figure 5b). 1) CdCl_2_–OcAm*_x_* complexes with the lamellar‐assembled structure are organized 2) Upon heating, the initial lamellar structure undergoes deformation and reacts with Se precursors. 3) As the reaction proceeds, small seeds of 2D nuclei are produced with halide ion binding, facilitating 2D growth along the lateral direction. The lateral dimension continuously expands along the side facets, which is more pronounced with the increased concentration of Cl^−^ ions. 4) During the nanosheet formation, the lamellar structures are reconstructed owing to the van der Waals interaction between nanosheets.

The difference in the precursor reactivity leads to two distinct synthesis routes. With highly reactive precursors, a large number of magic‐sized (CdSe)_13_ clusters assemble into 2D lamellar structures and then transform into 2D nanocrystals while maintaining 2D assemblies, resulting in the synthesis of nanoribbons. However, precursors with mild reactivity causes the formation of nanosheets to bypass the formation of nanocluster‐lamellar assemblies. Instead, Cl^−^ ions can facilitate 2D growth in the lateral direction and control the size of the final products. Our study shed light on the mechanistic pathways of the formation of quantum‐sized 2D CdSe nanocrystals.

## 4 Experimental Section

### Materials

Cadmium chloride (CdCl_2_, 99.99%), selenium (Se, 99.99%), *n*‐octylamine (OcAm, 99%), trioctylphosphine (TOP, 97%), chloroform (anhydrous, ≥99%), oleylamine (99%), 1‐octadecene (technical grade, 90%), and octane (anhydrous, ≥99%) were purchased from Sigma Aldrich. Ethyl alcohol (anhydrous, 99.9%) was purchased from Samchun Chemicals. *n*‐Octylammonium hydrochloride was purchased from Tokyo Chemical Industry. CO gas (99.999%) was purchased from Sumitomo Seika Chemicals.

### Synthesis of Wurtzite‐CdSe Nanoribbons

2D CdSe nanoribbons were synthesized by following a previously reported method with suitable modifications.^[^ ^23^ ^]^ The entire synthesis process of nanoribbons was conducted under an Ar atmosphere using standard Schlenk techniques. First, CdCl_2_–OcAm*_x_* complexes were synthesized by heating CdCl_2_ (1.5 mmol) in OcAm (10.0 mL) at 120 °C for 2 h. Next, *n*‐octylammonium selenocarbamate was prepared by bubbling CO gas into OcAm (5.0 mL) containing Se powder (4.5 mmol) under vigorous stirring for 2 h at room temperature. The resulting turbid white *n*‐octylammonium selenocarbamate (5.0 mL) was injected into the CdCl_2_–OcAm*_x_* (0 ≤ *x* ≤ 2) complex solution at 25 °C. The color of this reaction mixture gradually changed to light yellow and then white, suggesting the formation of nanoclusters. The solution was heated to 70 °C and maintained at this temperature for 24 h, following which it exhibited a turbid yellow color. The nanoribbons were precipitated using ethanol that containing TOP to remove unreacted Se precursor. The final products were obtained by several centrifugation and dispersion steps using pure ethanol. For isolating free (CdSe)_13_ clusters, as‐synthesized (CdSe)_13_ clusters were re‐dispersed in 14.0 mL of octane containing oleylamine (1.0 mL) and vigorously stirred for 3 days.

### Synthesis of Wurtzite‐CdSe Nanosheets

2D CdSe nanosheets were synthesized as per the previous method with suitable modifications.^[^ ^25^ ^]^ The entire synthesis process of nanosheets was conducted under an Ar atmosphere using standard Schlenk techniques. First, CdCl_2_ (1.5 mmol) was dissolved in OcAm (10.0 mL) and heated at 120 °C for 2 h to form CdCl_2_–OcAm*_x_* complexes. The Se precursor was prepared by mixing Se (4.5 mmol) and OcAm (5.0 mL) at room temperature. The Se precursor was injected into the CdCl_2_–OcAm*_x_* complex solution at 25 °C, and the mixture was heated at 100 °C for 24 h, during which the color of the reaction mixture changed from black to turbid yellow. The resultant product was washed with ethanol‐containing TOP to remove unreacted Se precursor. The final products were obtained by several centrifugation and dispersion steps using pure ethanol.

For the synthesis of CdSe nanosheets with additional Cl^−^ ions, CdCl_2_ (1.5 mmol) was dissolved in OcAm (10.0 mL) and heated at 120 °C for 2 h to form CdCl_2_–OcAm*_x_* complexes. Then, *n*‐octylammonium hydrochloride (3.0, 6.0, 12.0, and 27.0 mmol) was introduced to the CdCl_2_–OcAm*_x_* solution. Finally, the Se precursor was introduced to the above reaction solution at 25 °C, and the mixture was heated at 100 °C for 24 h. All the procedures were same as the synthesis of CdSe nanosheets except the initial quantity of Cl^−^ ions.

For the thermal stability test of CdSe nanosheets, after the complete formation of CdSe nanosheets by the reaction at 100 °C for 24 h, the reaction mixture was further heated to 160 °C and held at this temperature for 15 min. Then, the mixture was cooled to 25 °C for further analysis.

### Characterization

Absorption spectra were recorded using a Cary 5000 UV–vis–NIR spectrophotometer (Agilent Technologies). The PL spectra were obtained using a Fluoromax‐4 spectrofluorometer (Horiba). TEM images were obtained using Tecnai G2 F20 TWIN TMP (FEI). AFM images were collected using Park XE7 (Park Systems).

### In Situ SAXS Analysis

In situ SAXS measurements were performed at the PLS‐II 9A beamline of the Pohang Accelerator Laboratory (PAL) in Korea. The detector model was MX170‐HS (Rayonix). The beam‐exposure time (and integration time) was 5 s for each measurement and the X‐ray beam energy was 19.81 keV. The sample‐to‐detector distance was set as 2.0 m and the wave vector was ranging from 0.01 to ≈0.6 Å^−1^. 1D scattering spectra were obtained by azimuthally averaging 2D scattering images. The overall setup for the in situ measurements is depicted in Figure S4 (Supporting Information).

A small amount of the reaction mixture was loaded into a capillary and sealed with paraffin film. The capillary was then immediately placed on a heating holder, and the scattering patterns were recorded. The reaction mixtures were prepared by following the same protocol as those used for the syntheses of nanoribbons and nanosheets. The reaction mixtures, containing both CdCl_2_–OcAm*_x_* (0 ≤ *x* ≤ 2) complexes and Se precursor, were loaded into the capillary as the starting materials of the measurement. The amount of Se precursor was reduced to Cd:Se = 1:1 to avoid undesired scatterings. For wurtzite‐CdSe nanoribbons, magic‐sized (CdSe)_13_ clusters were already generated because of the high reactivity of the Se precursor. The time intervals for the measurements were 60 and 13 s for the nanoribbons and nanosheets, respectively.

A scattering pattern of the capillary containing pure OcAm solvent was taken and used as the background. SAXS data were analyzed after background subtraction. Background subtraction was processed by the following equation,^[^ ^79^ ^]^

(1) $$I\left(q\right)\;=\frac{C\left(q\right)-C_{d}\left(q\right)}{T\left(\lambda \right)t}-\;\frac{C_{b}\left(q\right)\;-\;C_{d}\left(q\right)}{T_{b}\left(\lambda \right)t_{b}}$$

where *C*(*q*) is the detected scattering intensity, *C*_b_(*q*) is the scattering of background, *C*_d_(*q*) is a dark current. *T*(*λ*) is the absorption, *t* is exposure time. *T*_b_(*λ*) and *t*_b_ are absorption and exposure times of background, respectively.

### In Situ Optical Spectroscopy

In situ absorption spectra were recorded using Lambda 465 UV–vis spectrometer (PerkinElmer) equipped with a fiber optic probe. The fiber was placed into a reaction pot containing CdCl_2_–OcAm*_x_* complexes. The reaction pot was then purged with Ar gas and sealed. Then, the Se precursor was injected into the reaction pot. All the synthesis conditions were the same as those used for the synthesis of nanosheets described above, except for the solution concentration to avoid signal saturation during the measurement. Ex situ absorption spectra (Figure S22, Supporting Information) were recorded for the synthesis of nanosheets at higher Cd concentrations (≥ 50 mm). In addition, the amount of Se precursor was reduced to Cd:Se = 1:1 to avoid undesired scatterings. The time interval for each measurement was set as 10 s. For the synthesis of nanoribbons, ex situ absorption spectra (in chloroform, with a dilution factor of ≈30) were recorded to avoid light scatterings (Figure S12, Supporting Information).

### Statistical Analysis

For the size distribution analysis of nanosheets and nanoribbons, TEM images were analyzed using ImageJ 1.53t software. The width and length were estimated based on the end‐to‐end distance of the nanocrystals. The number of samples (*n*) was 200.

## Conflict of Interest

The authors declare no conflict of interest.

## Supporting information

Supporting Information

## Data Availability

The data that support the findings of this study are available in the supplementary material of this article.
