# Supplementary material for: Revealing Two Distinct Formation Pathways of 2D Wurtzite‐CdSe Nanocrystals Using In Situ X‐Ray Scattering
Source: Adv Sci (Weinh). 2023 Dec 10;11(6):2307600. doi: 10.1002/advs.202307600 (PMC10853705; doi:10.1002/advs.202307600)
Supplement: Supplementary file 1 — Supporting Information [file ADVS-11-2307600-s001.pdf]

ADVANCED SCIENCE
Open Access

Supporting Information

for *Adv. Sci.*, DOI 10.1002/advs.202307600

Revealing Two Distinct Formation Pathways of 2D Wurtzite-CdSe Nanocrystals Using In Situ X-Ray Scattering

Hyo Cheol Lee, Megalamane S. Bootharaju, Kyunghoon Lee, Hogeun Chang, Seo Young Kim, Eonhyoung Ahn, Shi Li, Byung Hyo Kim, Hyungju Ahn, Taeghwan Hyeon* and Jiwoong Yang**

Supporting Information

Revealing Two Distinct Formation Pathways of 2D Wurtzite-CdSe Nanocrystals Using In-Situ X-ray Scattering

Hyo Cheol Lee, Megalamane S. Bootharaju, Kyunghoon Lee, Hogeun Chang, Seo Young Kim, Eonhyoung Ahn, Shi Li, Byung Hyo Kim, Hyungju Ahn, Taeghwan Hyeon*, and Jiwoong Yang**

H. C. Lee, K. Lee, S. Y. Kim, E. Ahn, S. Li, Prof. J. Yang
Department of Energy Science and Engineering, Daegu Gyeongbuk Institute of Science and Technology (DGIST), Daegu 42988, Republic of Korea
*E-mail: jiwoongyang@dgist.ac.kr

Dr. M. S. Bootharaju, Dr. H. Chang, Prof. B. H. Kim, Prof. T. Hyeon
Center for Nanoparticle Research, Institute for Basic Science (IBS), Seoul 08826, Republic of Korea
*E-mail: thyeon@snu.ac.kr

Dr. M. S. Bootharaju, Dr. H. Chang, Prof. T. Hyeon
School of Chemical and Biological Engineering, and Institute of Chemical Processes, Seoul National University, Seoul 08826, Republic of Korea

Dr. H. Chang
Samsung Advanced Institute of Technology, Samsung Electronics, Suwon 16678, Republic of Korea

Prof. B. H. Kim
Department of Material Science and Engineering, Soongsil University, Seoul 06978, Republic of Korea

Dr. H. Ahn
Pohang Accelerator Laboratory, Pohang 37673, Republic of Korea
*E-mail: hyungju@postech.ac.kr

Prof. J. Yang
Energy Science and Engineering Research Center, Daegu Gyeongbuk Institute of Science and Technology (DGIST), Daegu 42988, Republic of Korea

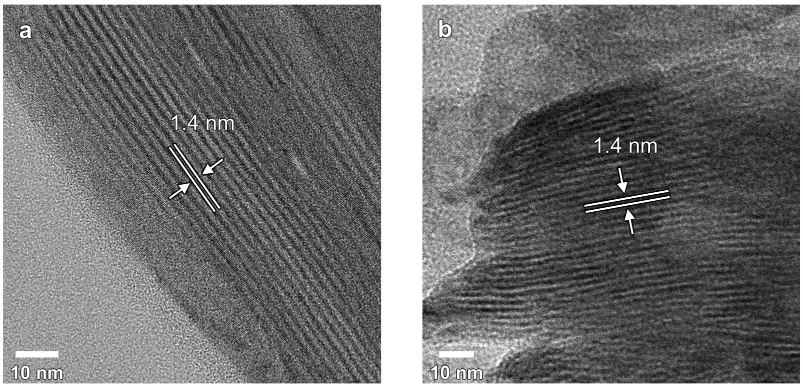

Figure S1. Side-view HR-TEM images of (a) nanoribbons and (b) nanosheets.

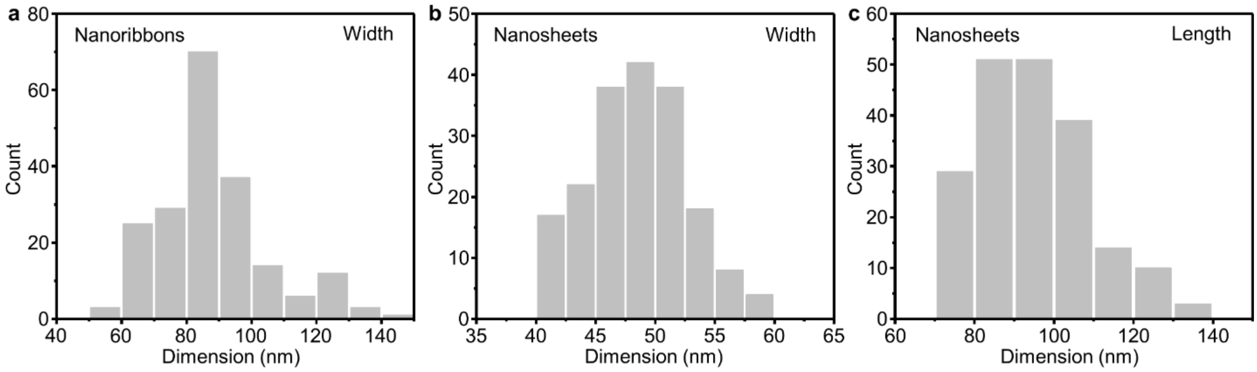

Figure S2. Size distributions of (a) width of CdSe nanoribbons, and (b) width and (c) length of CdSe nanosheets (n = 200).

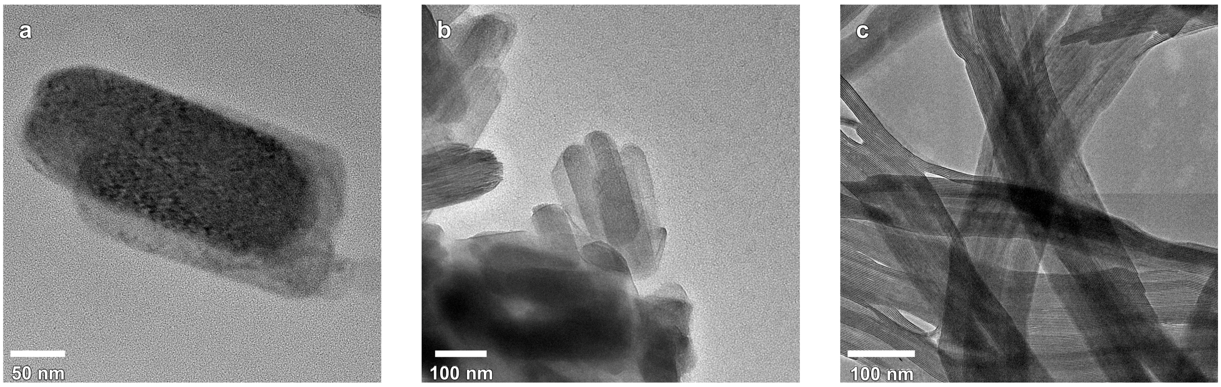

Figure S3. TEM images of (a, b) CdSe nanosheets and (c) CdSe nanoribbons.

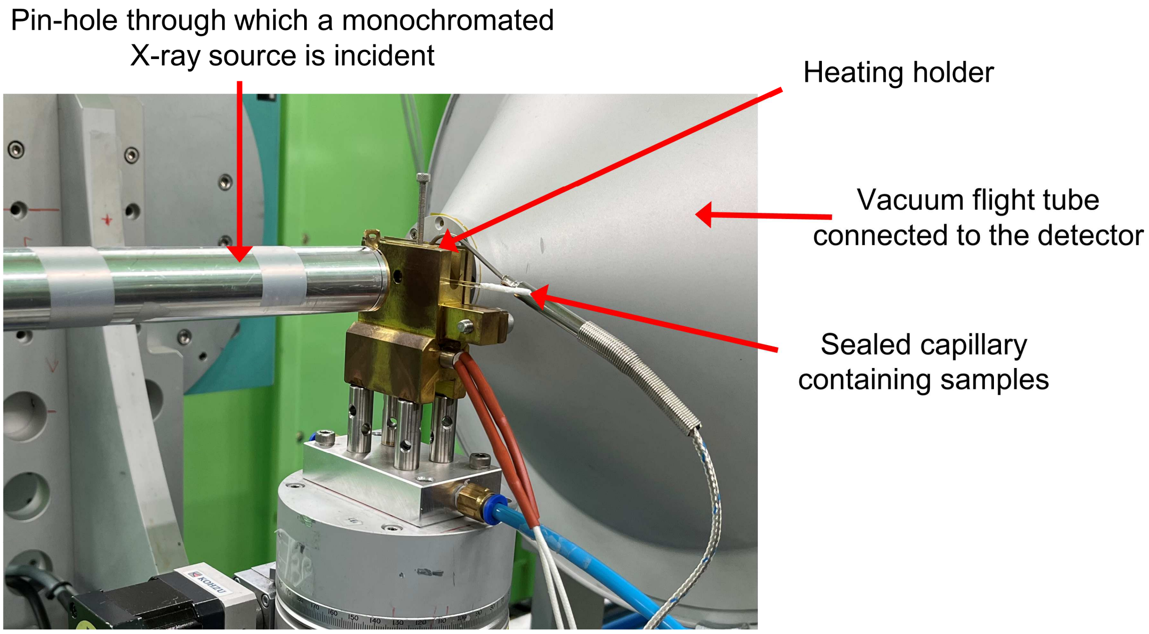

Figure S4. Photograph of the experimental setup for the *in-situ* SAXS measurements.

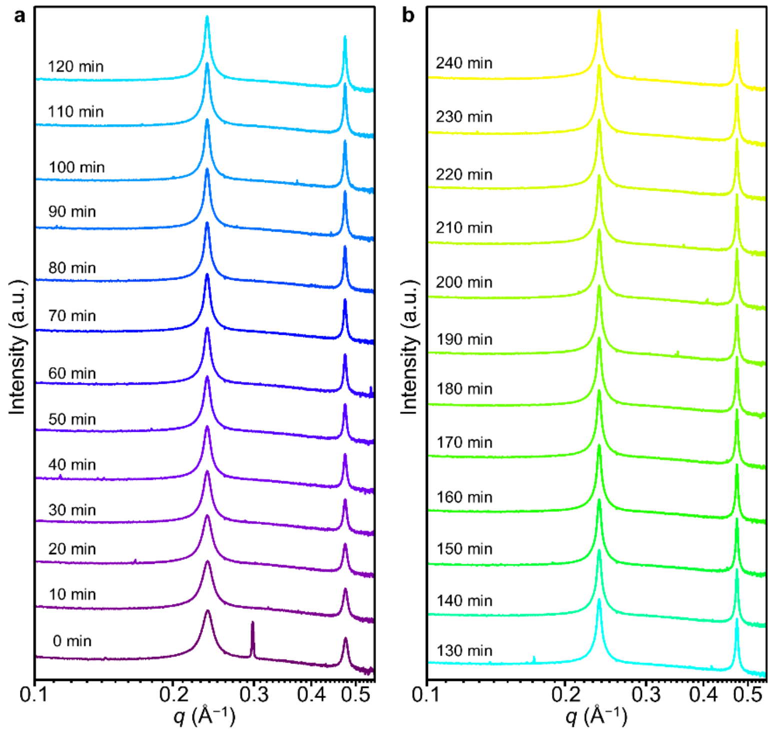

Figure S5. Representative *in-situ* SAXS patterns in Figure 2a, depicting the formation of CdSe nanoribbons at every 10 min for the first half of the reaction (0–4 h). The scattering patterns recorded for (a) 0–120 min and (b) 130–240 min of the reaction. The intensity is plotted using a logarithmic scale.

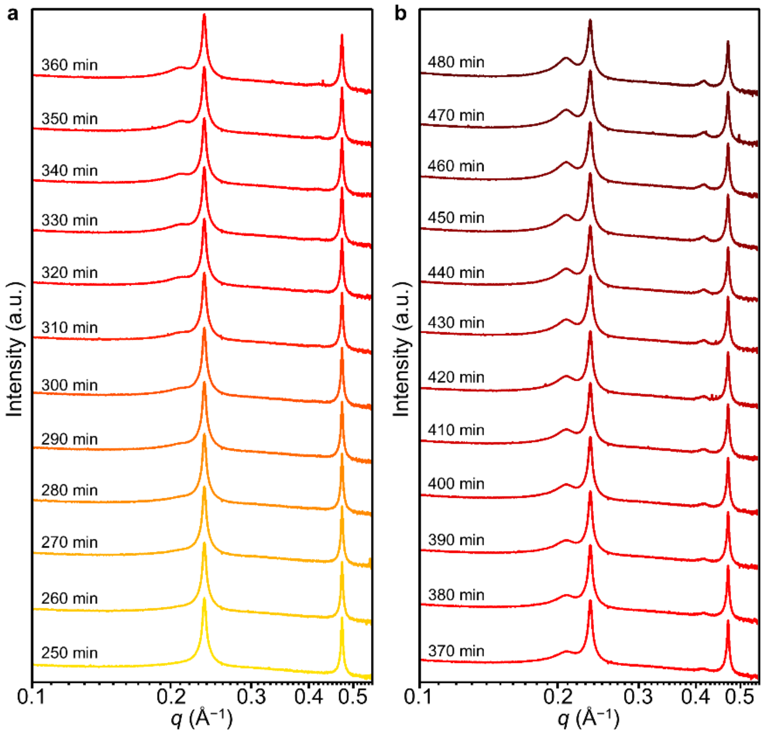

Figure S6. Representative *in-situ* SAXS patterns in Figure 2a, depicting the formation of CdSe nanoribbons at every 10 min for the latter half of the reaction (4–8 h). The scattering patterns recorded for (a) 250–360 min and (b) 370–480 min of the reaction. The intensity is plotted using a logarithmic scale.

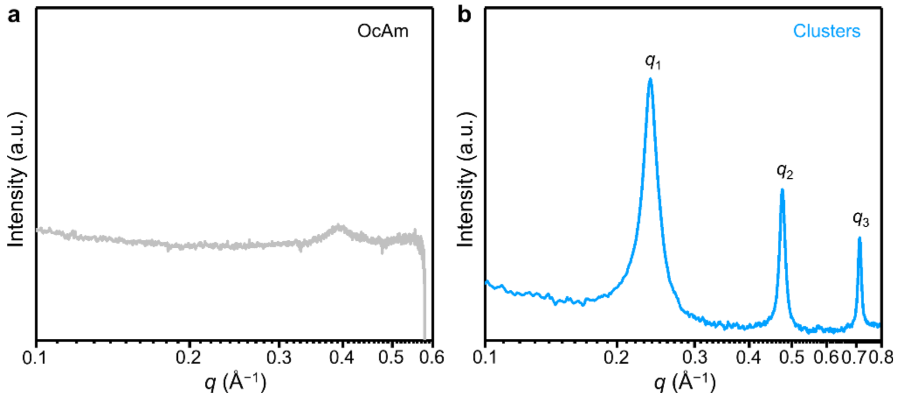

Figure S7. *Ex-situ* SAXS patterns of (a) the background OcAm solvent, and (b) the $(CdSe)_{13}$ clusters, showing a series of peaks. The positions of the first (q_1), second (q_2), and third (q_3)-order reflections are ~0.24, 0.48, and 0.71 Å^{-1}, respectively (q_1:q_2:q_3 = 1:2:3). The SAXS signal is compatible with a platelet-like stacking because no other peaks except the $q = n \times 0.24$ Å^{-1} are visible, where n is an integer number. The intensity is plotted using a logarithmic scale.

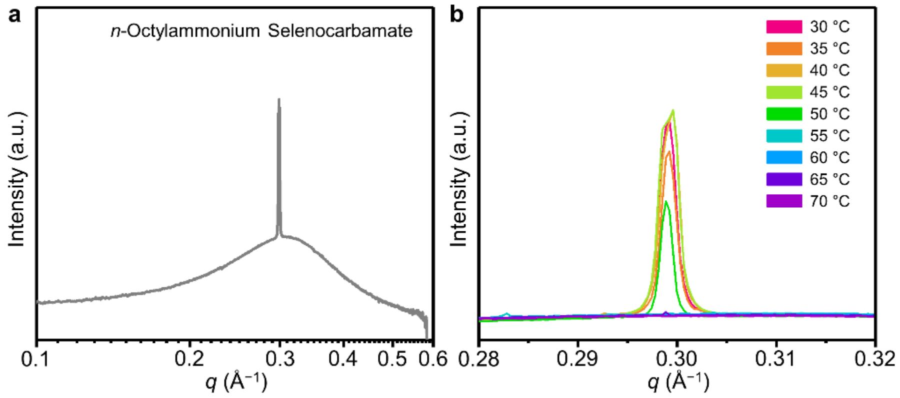

Figure S8. (a) *Ex-situ* SAXS pattern of *n*-octylammonium selenocarbamate in *n*-octylamine (concentration = 0.9 M Se). (b) The temperature dependent SAXS patterns of *n*-octylammonium selenocarbamate. The intensity is plotted using a logarithmic scale. Highly ionic characteristics of selenocarbamate attract and assemble *n*-octylamine at room temperature. Upon heating, the peak disappeared because of the dissociation of the assembly by thermal energy.

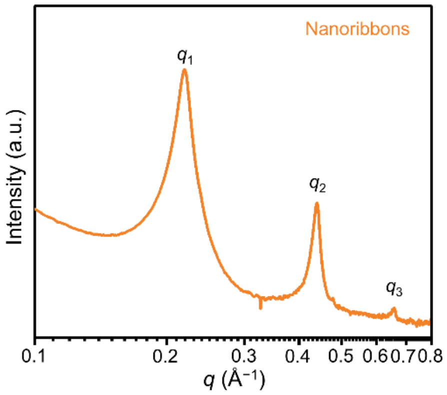

Figure S9. *Ex-situ* SAXS pattern of CdSe nanoribbons, showing a series of peaks. The positions of the first (q_1), second (q_2), and third (q_3)-order reflections are ~0.21, 0.43, and 0.65 Å^{-1}, respectively (q_1:q_2:q_3 = 1:2:3). The SAXS signal is compatible with a platelet-like stacking because no other peaks except the $q = n \times 0.21$ Å^{-1} are visible, where n is an integer number. The intensity is plotted using a logarithmic scale.

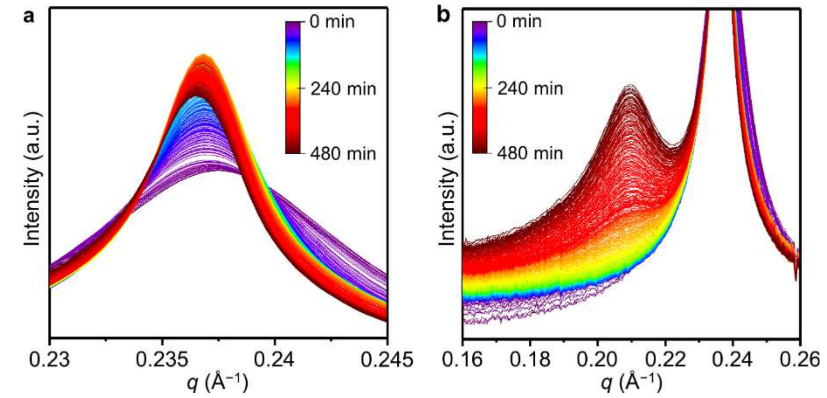

Figure S10. Magnified SAXS patterns of the regime corresponding to the first-order reflections of lamellar assemblies of (a) $(CdSe)_{13}$ clusters and (b) CdSe nanoribbons. The intensity is plotted using a logarithmic scale.

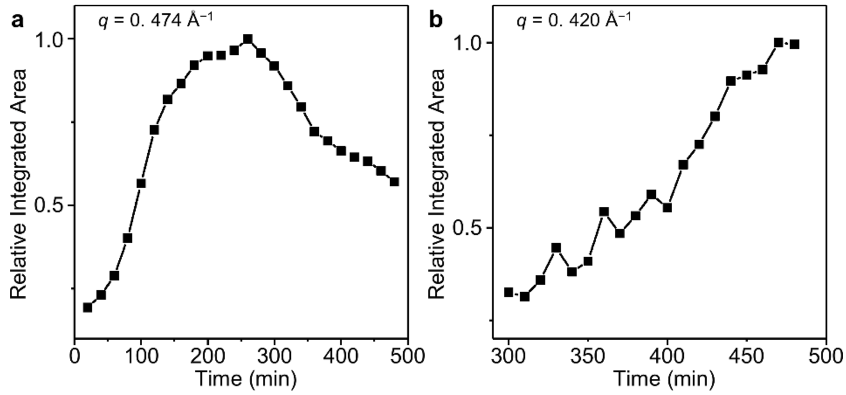

Figure S11. Relative integrated area for the second-order peaks of (a) the cluster assemblies (q = 0.474 Å^{-1}) and (b) the nanoribbon assemblies (q = 0.420 Å^{-1}) as a function of reaction time.

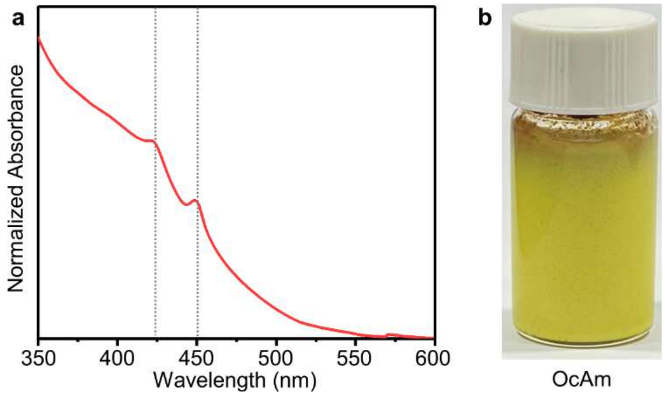

Figure S12. (a) Absorption spectrum (dilution factor = 10) and (b) photograph of nanoribbons dispersed in OcAm during the synthesis process. The gray dashed lines in panel a indicate the heavy hole- and light hole-excitonic transitions of nanoribbons.

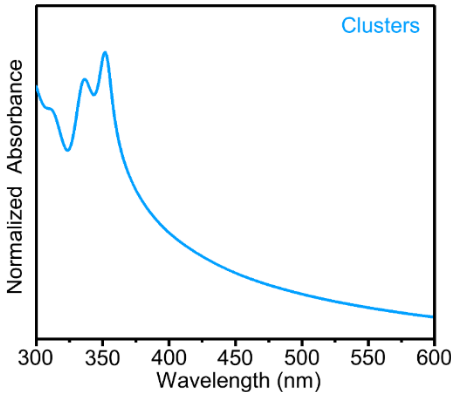

Figure S13. Absorption spectrum of $(CdSe)_{13}$ clusters.

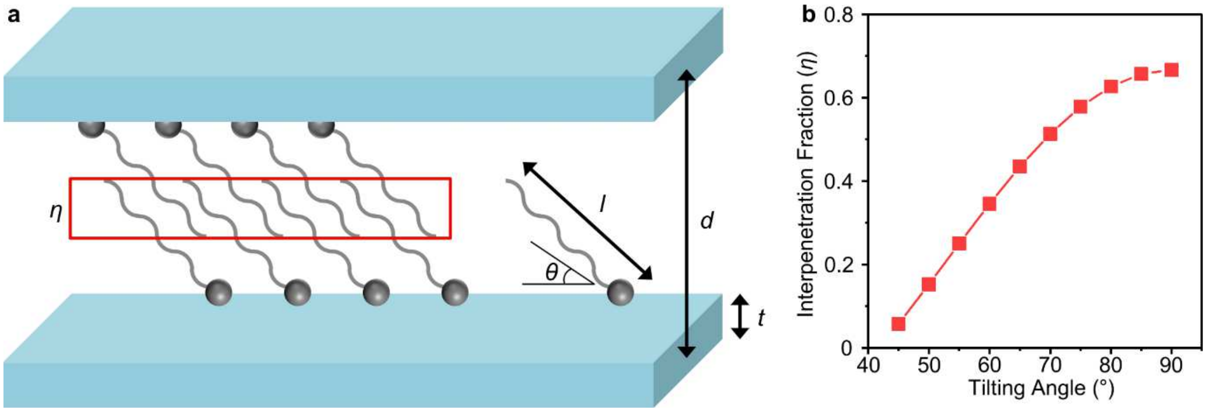

Figure S14. (a) Schematic illustration showing conformation and interdigitation of $(CdSe)_{13}$ lamellar assemblies. (b) Calculated interpenetration fraction (*η*) as a function of the tilting angle (*θ*). The thickness of lamellar assemblies (*t*) of clusters was estimated by TEM analysis (~1.1 nm) and the chain length (*l*) of OcAm was assumed as 1.2 nm.[S1] The *d*-spacing of 2.7 nm was used for the calculation.

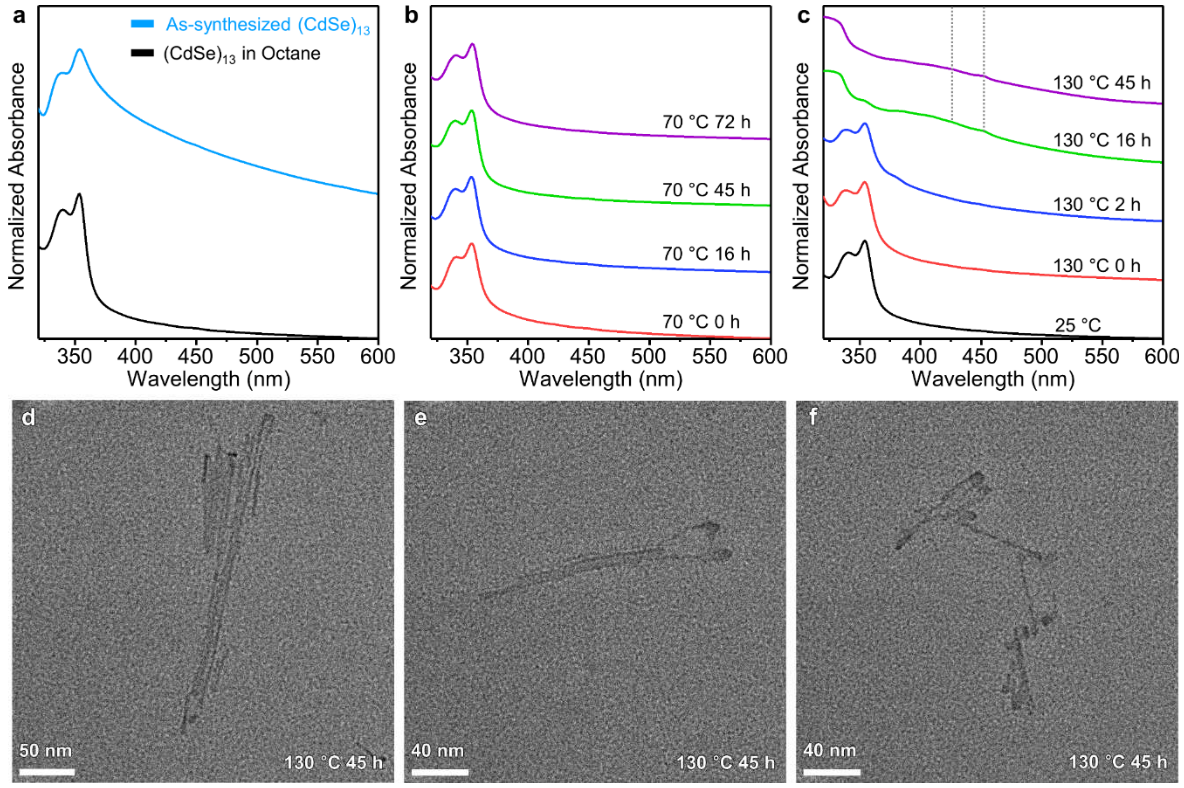

Figure S15. Control experiments using free $(CdSe)_{13}$ clusters. (a) Absorption spectra of as-synthesized and free $(CdSe)_{13}$ clusters dispersed in octane. The absorption spectrum of free $(CdSe)_{13}$ clusters shows no pronounced scattering tail, suggesting that the lamellar assemblies were disentangled. Absorption spectra of a series of aliquots during the heating of free $(CdSe)_{13}$ clusters (b) at 70 °C and (c) at 130 °C. The gray dashed lines indicate the heavy hole- and light hole-excitonic transitions of nanoribbons. For heating at 130 °C, 1-octadecene was used as the solvent. Free $(CdSe)_{13}$ clusters neither converted into nanoribbons nor dissolved at 70 °C. (d–f) TEM images of the products resulting from annealing of free $(CdSe)_{13}$ clusters at 130 °C for 45 h. Quasi 2D-like particles were obtained.

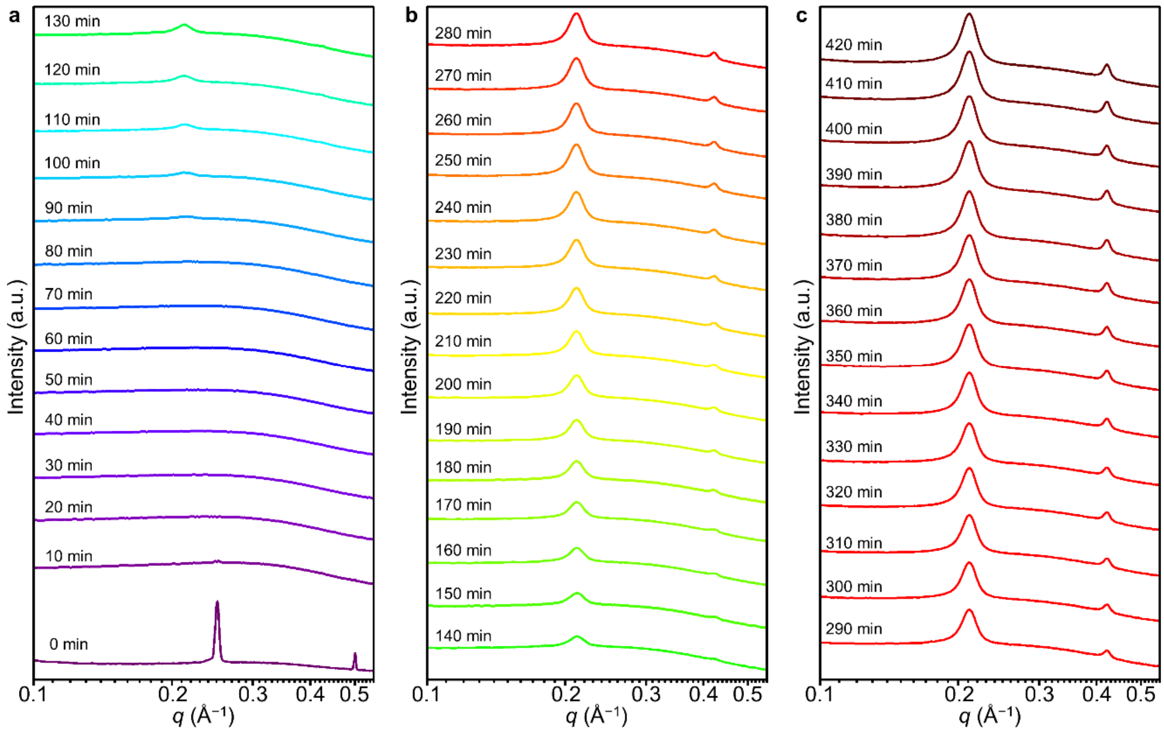

Figure S16. Representative *in-situ* SAXS patterns in Figure 3a, depicting the formation of CdSe nanosheets at every 10 min. The scattering patterns recorded for (a) 0–130, (b) 140–280, and (c) 290–420 min of the reaction. The intensity is plotted using a logarithmic scale.

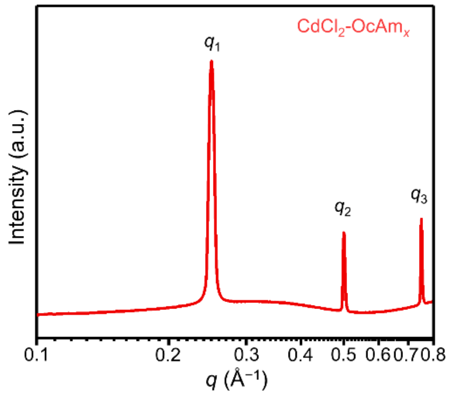

Figure S17. *Ex-situ* SAXS pattern of $CdCl_2$–$OcAm_x$ complexes, showing a series of peaks. The positions of the first (q_1), second (q_2), and third (q_3)-order reflections are ~0.25, 0.50, and 0.75 $Å^{-1}$, respectively (q_1:q_2:q_3 = 1:2:3). The intensity is plotted using a logarithmic scale.

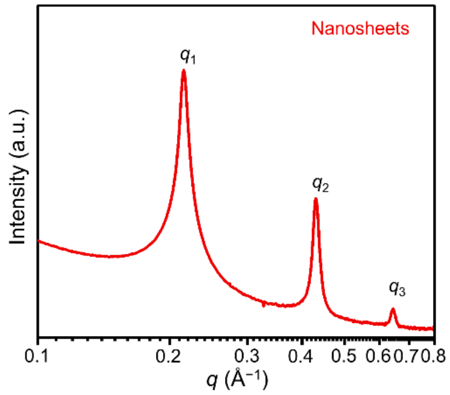

Figure S18. *Ex-situ* SAXS pattern of CdSe nanosheets, showing a series of peaks. The positions of the first (q_1), second (q_2), and third (q_3)-order reflections are ~0.21, 0.43, and 0.64 $Å^{-1}$, respectively (q_1:q_2:q_3 = 1:2:3). The SAXS signal is compatible with a platelet-like stacking because no other peaks except the $q = n \times 0.21$ $Å^{-1}$ are visible, where n is an integer number. The intensity is plotted using a logarithmic scale.

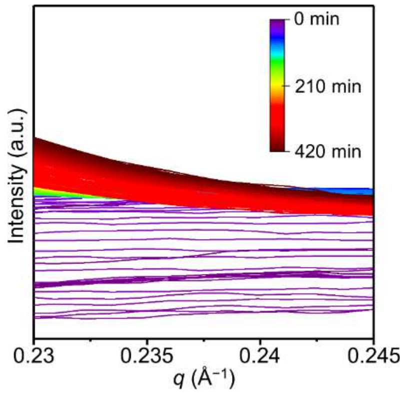

Figure S19. Magnified SAXS patterns on the regime corresponding to the first-order reflections of the lamellar assemblies of $(CdSe)_{13}$ clusters. The intensity is plotted using a logarithmic scale.

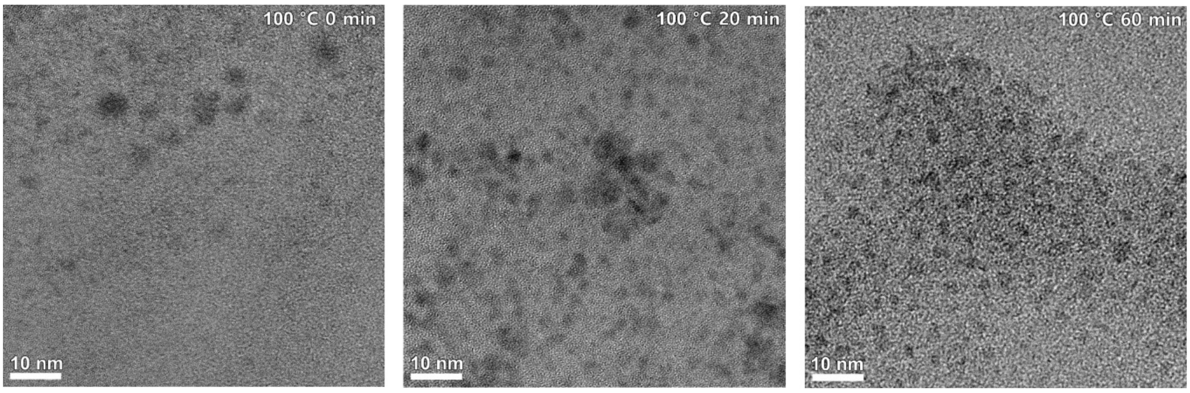

Figure S20. TEM analysis of aliquots withdrawn from the nanosheet synthesis. Both size and shape of particles (including small-sized nanosheets, 60 min) are highly irregular. Aliquots were taken during the synthesis and subsequently diluted with chloroform (dilution factor = 30) for the TEM analysis.

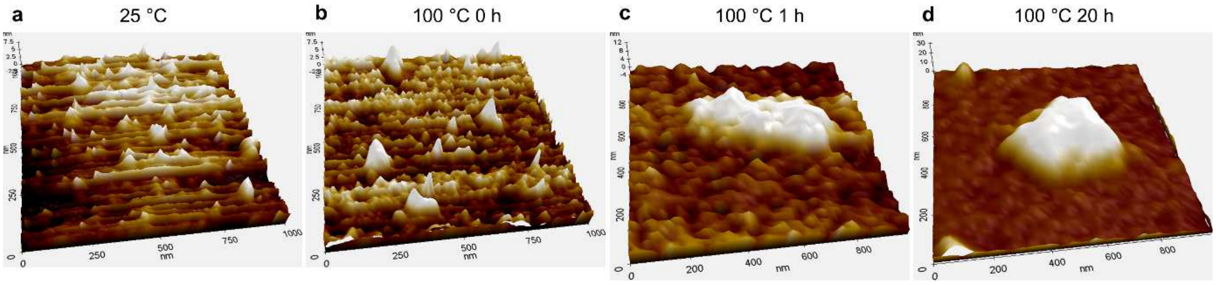

Figure S21. Atomic force microscopy analysis of the nanosheet synthesis at various growth stages: (a) at 25 °C, (b) 100 °C 0 h, (c) 100 °C 1 h, and (d) 100 °C 20 h.

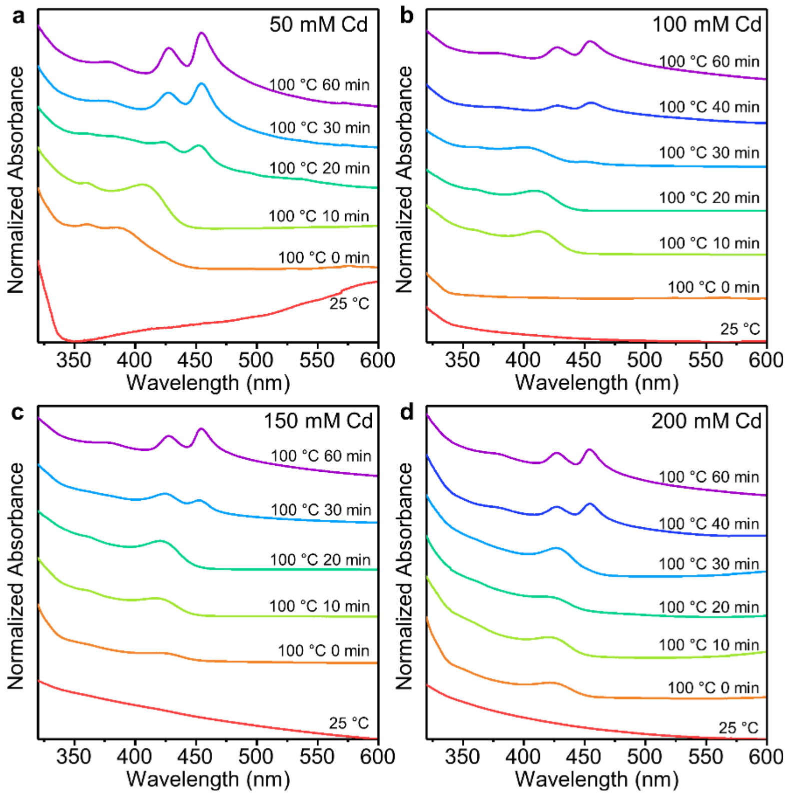

Figure S22. Temporal evolution of absorption spectra during nanosheet synthesis at different Cd precursor concentrations of (a) 50, (b) 100, (c) 150, and (d) 200 mM, which is equivalent to 0.5, 1.0, 1.5, and 2.0 times the initial concentration used for the nanosheet synthesis. The red line represents the sample acquired immediately after the injection of the Se precursor into the $CdCl_2$–$OcAm_x$ complexes.

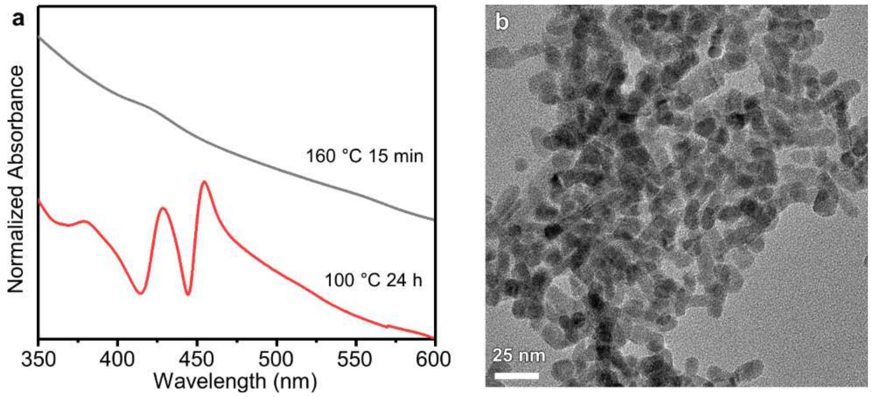

Figure S23. Thermal stability of lamellar nanosheets. (a) Absorption spectra of CdSe nanosheets before (red line) and after (gray line) heating to 160 °C and maintaining the temperature for 15 min. The heavy and light hole-excitonic transitions of the nanosheets disappeared, and a featureless spectrum appeared. (b) TEM image of nanosheets after heating. The 2D structures were fully destroyed, and dot or rod-like nanocrystals were synthesized.

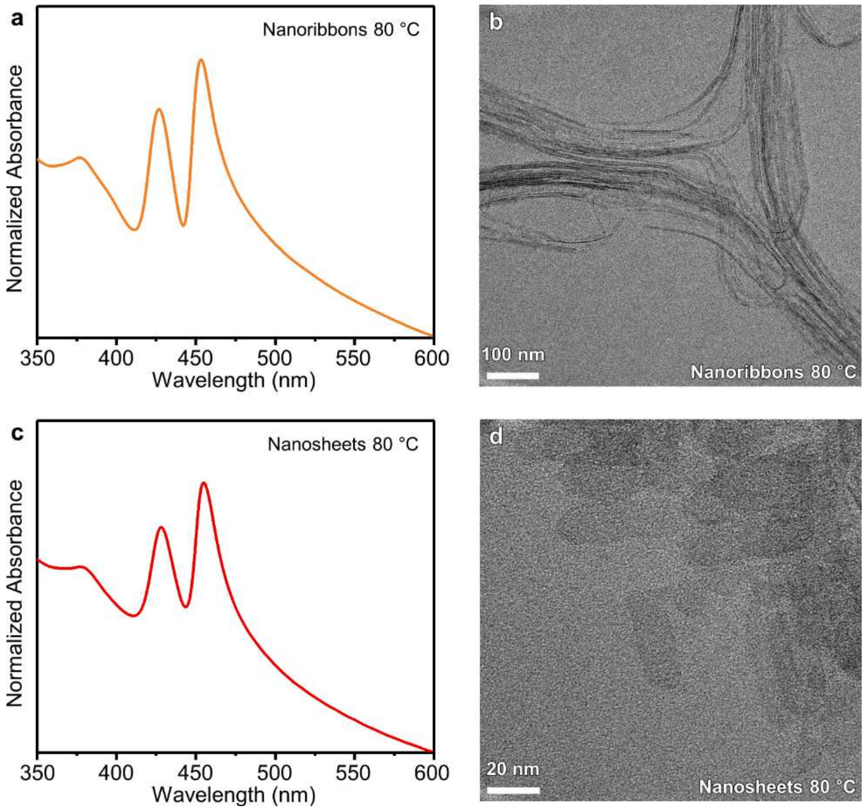

Figure S24. (a) Absorption spectrum and (b) TEM image of nanoribbons synthesized at 80 °C. (c) Absorption spectrum and (d) TEM image of nanosheets synthesized at 80 °C. All experimental parameters were maintained at their original values except for the reaction temperature.

References for the Supporting Information

[S1] Z. Wang, X.-D. Wen, R. Hoffmann, J. S. Son, R. Li, C.-C. Fang, D.-M. Smilgies, T. Hyeon, *Proc. Natl. Acad. Sci. U. S. A.* **2010**, *107*, 17119.
